# Career aspirations and factors influencing career choices of optometry students in Ghana

**DOI:** 10.1371/journal.pone.0233862

**Published:** 2020-05-29

**Authors:** Emmanuel Kobia-Acquah, Ebenezer Owusu, Kwadwo Owusu Akuffo, Nana Yaa Koomson, Tchiakpe Michel Pascal

**Affiliations:** Department of Optometry and Visual Science, Kwame Nkrumah University of Science and Technology, Private Mail Bag, University Post Office, Kumasi, Ghana; University of Lincoln, UNITED KINGDOM

## Abstract

Optometry students in clinical years are usually faced with the challenges of making a career choice in or outside optometry. This cross sectional study was conducted to investigate the career aspirations of optometry students in Ghana. All students in their fourth to sixth year who consented to participate in the study responded to a questionnaire which explored: demographic characteristics, career aspirations, and factors influencing their choices. Descriptive statistics was used to present data as frequencies, proportions and percentages. Chi-square test and logistic regression analysis were used to evaluate relationships between variables. Two hundred and nine students from the two optometry training institutions in Ghana; Kwame Nkrumah University of Science and Technology (49.8%) and University of Cape Coast (51.2%) responded to the questionnaire. The mean (SD) age of students was 23.6 (1.9) years (males = 65.6%). On seeking admission into the university, optometry (65.6%) and human biology/medicine (28.2%) were the leading first choice programmes among participants. Participants largely aspire to be in clinical practice (64.6%) or Academia/Research (28.2%). The major factors which influenced career choices were interest in career field (64.1%) and potential good income (38.3%). Females were twice more likely to practice optometry and pursue an interest in paediatric optometry than males. Institution of study (p = .028) and information on career opportunities (p = .018) were significant predictors of students’ decision to pursue a career in academia/research. Optometry students in Ghana largely aspire to be in clinical practice, a finding which is useful for optometry training institutions and relevant stakeholders in developing the optometry programme and projecting its future in Ghana.

## Introduction

Career decisions play a crucial role in an individual’s chosen profession and overall success in life. Wrong career decisions can have negative effects in an individual’s life so most people attach some degree of diligence in selecting a career option. However, there is a complex interplay of factors such as a person’s psychosocial make-up, societal expectations, job availability and personal ethos that can influence an individual’s career decision [1–4]. Career choices of health professionals such as optometrists do not affect only the individual but the society and country at large, because these decisions can go a long way to affect human resource for healthcare delivery in the country. In developing countries where there is a shortage of health personnel, these can have dire consequences on the overall health care delivery in the country [5].

Optometry as a profession and optometric education in Africa have progressed steadily with an increasing number of optometry schools being established across the continent in recent years. Optometry degree in Africa is run as either a six-year degree programme leading to the award of a Doctor of Optometry (OD) degree or four-year degree programme after which a Bachelor of Optometry degree (BOptom) is awarded [6–8]. The programme is currently offered in only two institutions in Ghana, the Kwame Nkrumah University of Science and Technology (KNUST) and University of Cape Coast (UCC), leading to the award of an OD degree. Optometrists are now considered significant health personnel in the eye care delivery system largely due to their role in treating uncorrected refractive error, the leading cause of visual impairment globally [5,9]. The services of optometrists in rural areas of the country have become increasingly important due to the lack of ophthalmologists in these areas coupled with the improved training and expanded scope of practice of optometrists in Ghana. This means that optometrists do not only have to treat refractive error but also prescribe drugs to treat a wide range of anterior segment conditions [10,11]. As a result of these, the career choices of optometry students may have a direct impact on the burden of visual impairment and the country’s overall goal of achieving VISION 2020 and the Global Action Plan (GAP) Initiatives.

Optometry students in the clinical years are usually faced with difficulties of settling on a career in or outside optometry. Careers in optometry could be practice oriented (with or without further interest areas in optometry) or non-practice oriented (academia, research and industrial optometry). However, others in pursuit of their passions and interests may enter into entirely different fields such as humanities, medicine, business administration and law. Factors such as career opportunities, length of residency, and work-life-balance have been reported to have a significant impact on medical students’ choice of career [1,2,12]. A study conducted by Singh and Singh [13] in India on medical students’ career aspiration and their determinants revealed that a significant number of graduating doctors could pursue non-medical and commerce related fields after completing their bachelors; the central reasons being less challenging tasks, more economic stability at an earlier age and managing a family. Studies on career aspirations of health care practitioners have widely been conducted among medical students, however, there is limited evidence of motivation for choosing optometry as a career. In Saudi Arabia, major factors influencing optometry as a career choice included altruism, career prestige and the introduction of the new Doctor of Optometry programme that has more ocular disease and treatment focus [14]. The only study conducted in Ghana to profile optometry students found that ‘job availability after graduation’ and ‘desire to help other people’ were major reasons for choosing optometry [15]. The source and availability of information are also important and can influence an individual in making career decisions. Students’ exposure to practitioners in the fields of agriculture [16] and medicine [17] appeared to heighten their interest in those fields. High parental influence has also been reported to have an impact on career choices of medical students in the United Arab Emirates [18] and United States of America [19].

The career aspirations of optometry students in Ghana, though personal, have consequential effects on the country’s eye services delivery and the future of optometric education and its practice in Ghana. This study will corroborate or repudiate the earlier findings on factors influencing career choices of optometry students in Ghana, five years after the study was conducted. The previous study did not assess career fields and optometric interest areas that optometry students may aspire to pursue [15]. Given that Ghana has made significant strides in the training of optometrists over the years, with recently trained optometrists having access to newer technologies as well as being better informed on the different interest areas in optometry, it is important to assess whether motivations for pursuing a career in optometry have changed over the years. This will assist institutional heads in recruitment of prospective students and strengthen the career counselling approach for students. Also, the Ghana Optometric Association (GOA), Ministry of Health and other relevant stakeholders in eye care can rely on the findings for policy development on optometry and eye care in Ghana as the country makes projections for eye care delivery post-VISION 2020. The objective of this study was to investigate the career aspirations and associated factors among optometry students in Ghana.

## Materials and methods

This was a cross sectional survey using a self-developed questionnaire (S1 Table) which was pretested on ten third year KNUST optometry students and subsequently modified for easy understanding. Included in the study were all 215 students in fourth to sixth year who were registered for the 2018/2019 academic year at the two optometry schools in Ghana (KNUST and UCC). Only students in their clinical years (year 4–6) were included because they would have been more informed about various career opportunities to be able to response appropriately to the questionnaires. Participants answered self-administered questionnaires (S1 Table); data on participant’s demographics, choice of optometry programme, career aspirations, primary source of information on career options, and factors influencing career choices were analyzed.

Data entry and analysis were done using the Statistical Product and Service Solutions (IBM SPSS Inc., Chicago, Illinois, USA) Version 25. Confidence interval was set at 95% and p < .05 was considered statistically significant. The study population was described using descriptive statistics to present data as frequencies, proportions and percentages. A Chi-Square test and logistic regression analysis were used to evaluate relationships between variables.

The study adhered to the guidelines of the Declaration of Helsinki. Ethical approval for the study was obtained from the Committee on Human Research, Publications & Ethics of KNUST and Komfo Anokye Teaching Hospital, Kumasi. Permission was taken from the two heads of the Optometry department at KNUST and UCC and participants signed an informed consent to partake in the study.

## Results

### Participants demographics

A total of 209 students (97.2% response rate) responded to the questionnaire (51.2% from UCC and 48.8% from KNUST). The mean (SD) age of participants was 23.6 (1.9) years (65.6% were males). Almost all participants were Ghanaian students (98.6%) with majority from urban areas (61.2%). Participants were from all the ten administrative regions of Ghana with Ashanti Region having the highest representation (24.3%) and the Upper West Region (4.4%) least represented. The majority (36.8%) of respondents were in the fourth year (Table 1).

**Table 1:** Age and gender distribution by nationality, place of origin, institution, year of study, and Ghanaian region.

Characteristics	Up to 24 years		≥ 25 years		Total	
	Male n (%)	Female n (%)	Male n (%)	Female n (%)	Male n (%)	Female n (%)
**Nationality**						
Ghanaian	80 (58.4)	61 (84.7)	56 (40.9)	9 (12.5)	136 (99.3)	70 (97.2)
Non-Ghanaian	1 (0.7)	2 (2.8)	0 (0.0)	0 (0.0)	1 (0.7)	2 (2.8)
Total	81 (59.1)	63 (87.5)	56 (40.9)	9 (12.5)	137 (100.0)	72 (100.0)
**Place of Origin**						
Rural	28 (20.4)	19 (26.4)	30 (21.9)	1 (1.4)	58 (42.3)	20 (27.8)
Urban	53 (38.7)	44 (61.1)	26 (19.0)	8 (11.1)	79 (57.7)	52 (72.2)
Total	81 (59.1)	63 (87.5)	56 (40.9)	9 (12.5)	137 (100.0)	72 (100.0)
**Institution**						
KNUST	37 (27.0)	39 (54.2)	22 (16.1)	4 (5.6)	59 (43.1)	43 (59.7)
UCC	44 (32.1)	24 (33.3)	34 (24.8)	5 (6.9)	78 (56.9)	29 (40.3)
Total	81 (59.1)	63 (87.5)	56 (40.9)	9 (12.5)	137 (100.0)	72 (100.0)
**Year of Study**						
Year Four	45 (32.8)	29 (40.3)	3 (2.2)	0 (0.0)	48 (35.0)	29 (40.3)
Year Five	20 (14.6)	14 (19.4)	22 (16.1)	1 (1.4)	42 (30.7)	15 (20.8)
Year Six	16 (11.7)	20 (27.8)	31 (22.6)	8 (11.1)	47 (34.3)	28 (38.9)
Total	81 (59.1)	63 (87.5)	56 (40.9)	9 (12.5)	137 (100.0)	72 (100.0)
**Ghanaian Region**						
Ashanti	22 (16.2)	15 (21.4)	11 (8.1)	2 (2.9)	33 (24.3)	17 (24.3)
Northern	5 (3.7)	8 (11.4)	1 (0.7)	2 (2.9)	6 (4.4)	10 (14.3)
Brong Ahafo	7 (5.1)	4 (5.7)	1 (0.7)	0 (0.0)	8 (5.9)	4 (5.7)
Upper East	4 (2.9)	3 (4.3)	1 (0.7)	2 (2.9)	5 (3.7)	5 (7.1)
Eastern	11 (8.1)	11 (15.7)	8 (5.9)	1 (1.4)	19 (14.0)	12 (17.1)
Upper West	3 (2.2)	1 (1.4)	4 (2.9)	1 (1.4)	7 (5.1)	2 (2.9)
Greater Accra	3 (2.2)	8 (11.4)	6 (4.4)	1 (1.4)	9 (6.6)	9 (12.9)
Western	8 (5.9)	3 (4.3)	5 (3.7)	0 (0.0)	13 (9.6)	3 (4.3)
Volta	2 (1.5)	3 (4.3)	6 (4.4)	0 (0.0)	8 (5.9)	3 (4.3)
Central	15 (11.0)	5 (7.1)	13 (9.6)	0 (0.0)	28 (20.6)	5 (7.1)
Total	80 (58.8)	61 (87.1)	56 (41.2)	9 (12.9)	136 (100.0)	70 (100.0)

KNUST = Kwame Nkrumah University of Science and Technology; UCC = University of Cape Coast

### Initial programme of choice

Optometry was the first programme of choice for 65.6% of the students when they were seeking admission into the university followed by Human Biology/Medicine (28.2%), Pharmacy (4.3%), Engineering (1.4%) and Computer Science (0.5%). More than half (56.9%) of KNUST students chose optometry as their first programme of study compared to 73.8% of UCC students. UCC students were more likely to choose optometry as a first choice programme of study during university application (χ^2^ (1) = 6.659, p = .01).

### Career aspirations

**Fig 1:**
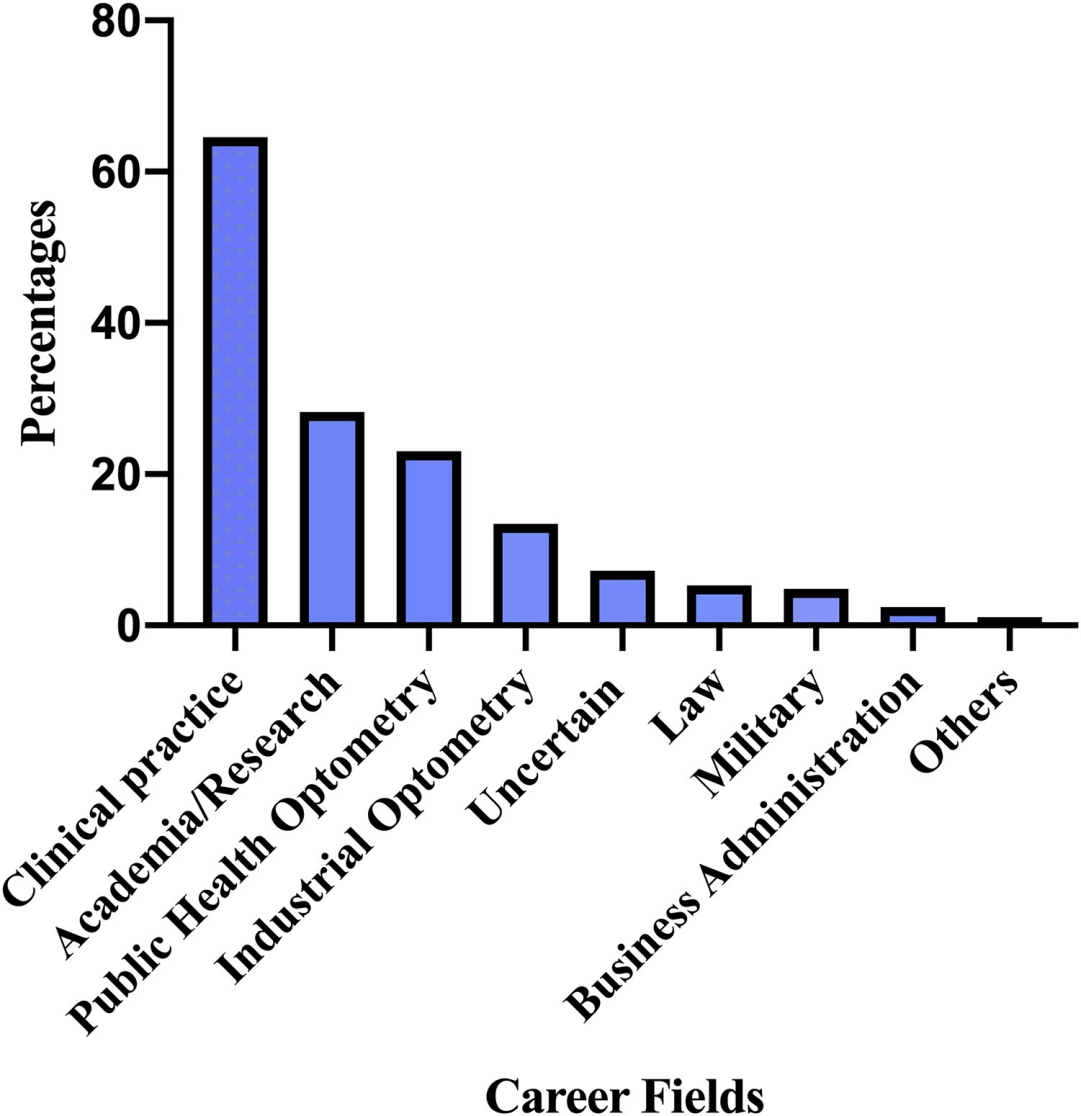
Career fields students aspire to pursue.

**Fig 2:**
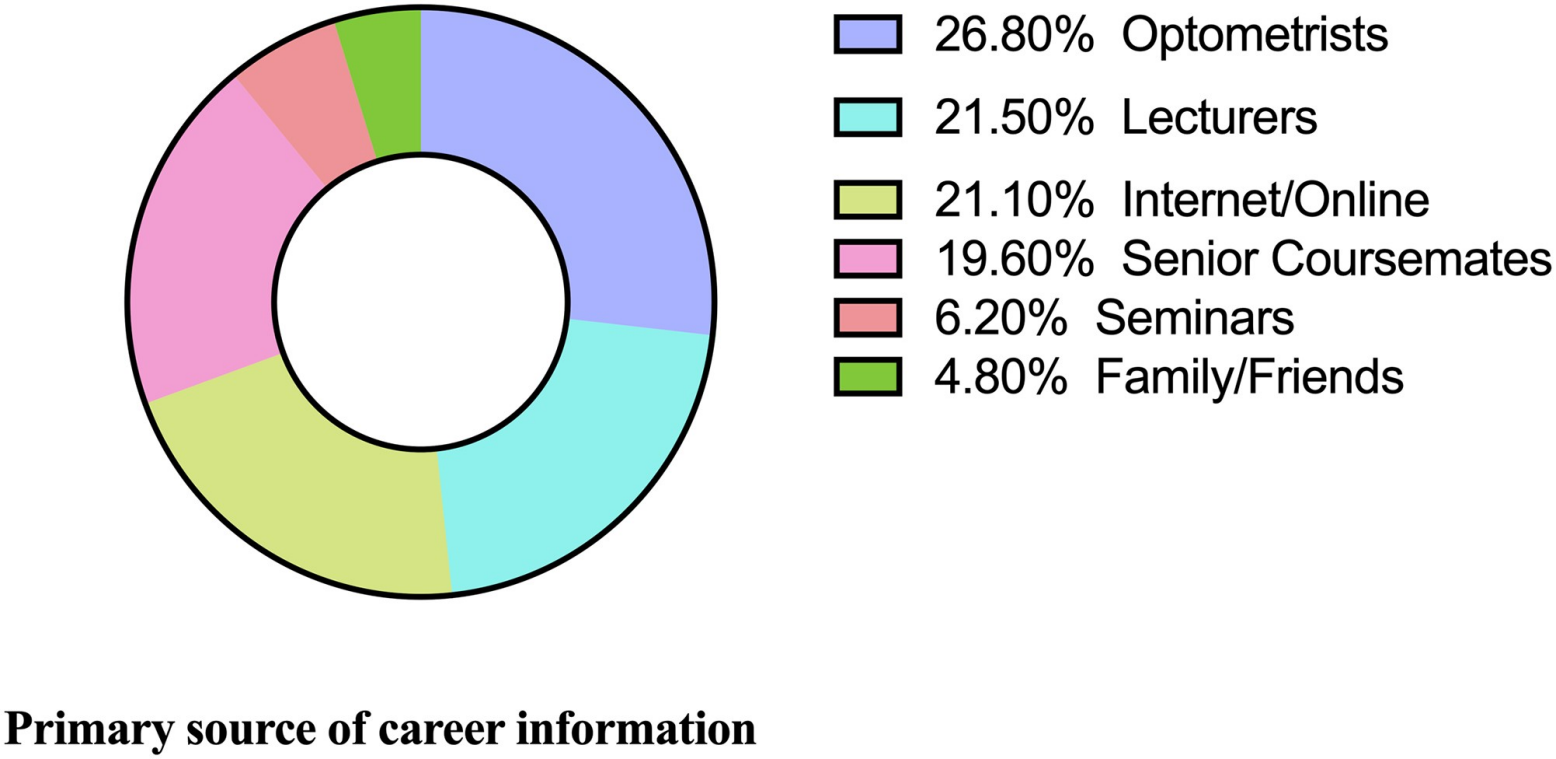
Primary source of career information.

The majority of participants aspired to be in clinical practice (64.6%) (Fig 1) and Environmental/Occupational optometry was the most desired optometric interest area (28.7%) (Table 2). Information from optometrists (26.8%) was the leading primary source of career information (Fig 2). There was no association between career aspirations and institution of study (Table 3) and participants’ choice of optometry as an initial programme of study (Table 4).

**Table 2:** Optometric interest areas students aspire to pursue.

Interest area*	Yes	Percentage (%)
	N = 209	
Contact lens	50	23.9
Low vision	52	24.9
Paediatric optometry	56	26.8
Geriatric optometry	35	16.7
Environmental/Occupational optometry	60	28.7
Binocular vision	49	23.4

*Participants gave multiple responses for choice of optometric interest area

**Table 3:** Career aspirations of students who chose optometry as first choice programme.

Career Choices*	Optometry n (%)	Other programmes n (%)	p-value
**Clinical Practice**				
No (74)	47 (63.5)	27 (36.5)	n.s
Yes (135)	90 (66.7)	45 (33.3)	
**Academia/Research**				
No (150)	98 (65.3)	52 (34.7)	n.s
Yes (59)	39 (66.1)	20 (33.9)	
**Industrial optometry**				
No (181)	120(66.3)	61 (33.7)	n.s
Yes (28)	17 (60.7)	11 (39.3)	
**Public health**			
No (161)	103 (64)	58 (36)	n.s
Yes (48)	34 (66.1)	14 (33.9)	
**Non optometric**				
No (181)	118 (65.2)	63 (34.8)	n.s
Yes (28)	19 (67.9)	9 (32.1)	
**Contact Lens**			
No (159)	105 (66)	54 (34)	n.s
Yes (50)	32 (64)	18 (36)	
**Low Vision**			
No (157)	106 (67.5)	51 (32.5)	n.s
Yes (52)	31 (59.6)	21 (40.4)	
**Paediatric optometry**				
No (153)	101 (66)	52 (34)	n.s
Yes (56)	36 (64.3)	20 (35.7)	
**Geriatric optometry**			
No (174)	117 (67.2)	57 (32.8)	n.s
Yes (35)	20 (57.1)	15 (42.9)	
**Environmental/Occupational optometry**			
No (149)	92 (61.7)	57 (38.3)	n.s
Yes (60)	45 (75)	15 (25)	
**Binocular Vision**			
No (160)	103 (64.4)	57 (35.6)	n.s
Yes (49)	34 (69.4)	15 (30.6)	

*Participants gave multiple responses; n (%) represents the number and percentages of participants respectively; Statistical significance was tested with chi-square with level of significance set at p<0.05; n.s = not significant

**Table 4:** Career aspirations of students’ in the two institutions of study.

Career Choices*	KNUST n (%)	UCC n (%)	p-value
**Clinical Practice**			
No (74)	34 (45.9)	40 (54.1)	n.s
Yes (135)	68 (50.4)	67 (49.6)	
**Academia/Research**			
No (150)	68 (45.3)	82 (54.7)	n.s
Yes (59)	34 (57.6)	25 (42.4)	
**Industrial optometry**			
No (181)	84 (46.4)	97 (53.6)	n.s
Yes (28)	18 (64.3)	10 (35.7)	
**Public health**			
No (161)	84 (52.2)	77 (47.8)	n.s
Yes (48)	18 (37.5)	30 (62.5)	
**Non optometric**			
No (181)	85 (47)	96 (53)	n.s
Yes (28)	17 (60.7)	11 (39.3)	
**Contact Lens**			
No (159)	75 (47.2)	84 (52.8)	n.s
Yes (50)	27 (54)	23 (46)	
**Low Vision**			
No (157)	76 (48.4)	81 (51.6)	n.s
Yes (52)	26 (50)	26 (50)	
**Paediatric optometry**			
No (153)	69 (45.1)	84 (54.9)	n.s
Yes (56)	33 (58.9)	23 (41.1)	
**Geriatric optometry**			
No (174)	90 (51.7)	84 (48.3)	n.s
Yes (35)	12 (34.3)	23 (65.7)	
**Environmental/Occupational optometry**			
No (149)	73 (49)	76 (51)	n.s
Yes (60)	29 (48.3)	31 (51.7)	
**Binocular Vision**			
No (160)	74 (46.2)	86 (53.8)	n.s
Yes (49)	28 (57.1)	21 (42.9)	

*Participants gave multiple responses; n (%) represents the number and percentages respectively of participants; Statistical significance was tested with chi-square with level of significance set at p<0.05; n.s = not significant

### Factors influencing career choices

**Fig 3:**
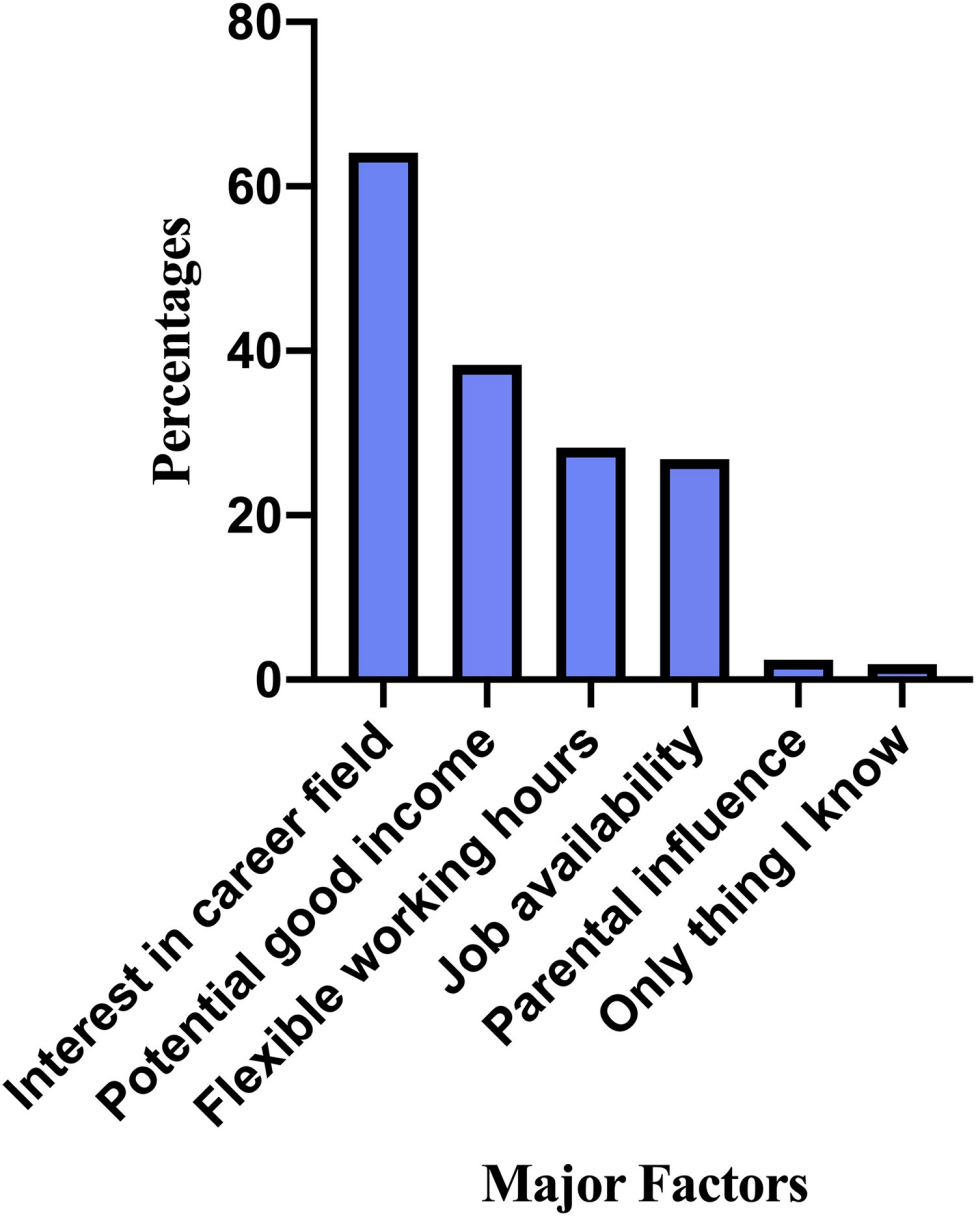
Major factors influencing career choices of participants.

Interest in career field (64.1%) was the major factor the students considered in making their career choices followed by potential good income (38.3%) (Fig 3). Binary logistic regression was performed to assess the impact of a number of demographic factors on the likelihood that students would pursue the top two career options; clinical practice and academia/research. The model contained eight independent variables; age, gender, institution, place of origin, year of study, initial choice programme during university application, information on career opportunities, and primary source of career information. Only gender made a significant contribution (p = .024) to the model and was a significant predictor of clinical practice with an odds ratio of 2.07 (Table 5). This indicated that female students were twice more likely to practice optometry than males, controlling for all other factors in the model. For academia/research, two factors (institution, p = .028 and information on career opportunities, p = .014) made significant contributions to the model. The strongest predictor of pursuing an academic/research career was information on career opportunities recording an odds ratio of 5.18 (Table 6). This indicated that students who had good/very good information on academia/research were approximately 5 times more likely to pursue academia/research than students who did not receive good information, controlling for all other factors in the model. Binary logistic regression was also performed to assess the impact of a number of demographic factors on the likelihood that students would pursue the top two optometric interest areas; environmental/occupational (Table 7) and paediatric optometry (Table 8). No significant associations were observed between environmental/occupational optometry and students’ demographic characteristics. For paediatric optometry, only gender made a signicant contribution (p = .012) to the model and was the strongest predictor of paediatric optometry with an observed odds ratio of 2.23 (Table 8). This indicated that females were approximately twice more likely to pursue paediatric optometry as an interest area than males, controlling for all other factors in the model.

**Table 5:** Logistic regression analysis of demographic factors associated with clinical practice.

Factors	Odds Ratio	95% C.I for Odds Ratio		p-value
		Lower Bound	Upper Bound	
Age	1.065	.901	1.258	.459
Gender	2.074	1.101	3.906	.024
Institution	.786	.421	1.465	.448
Place of origin	.761	.409	1.417	.389
Year of study	.847	.414	1.733	.650
First choice programme	.716	.384	1.337	.295
Career information	1.851	.774	4.425	.166
Source of career information	.369	.094	1.450	.153

χ^2^ = 5.359; *df* = 1; p = .021; C.I = Confidence Interval

**Table 6:** Logistic regression analysis of demographic factors associated with academia/research.

Factors	Odds Ratio	95% C.I for Odds Ratio		p-value
		Lower Bound	Upper Bound	
Age	1.082	.893	1.311	.422
Gender	.569	.277	1.168	.124
Institution	.479	.248	.924	.028
Place of origin	1.873	.943	3.722	.073
Year of study	.826	.385	1.775	.625
First choice programme	.922	.454	1.873	.823
Information on career opportunity	5.183	1.388	19.347	.014
Source of career information	1.188	.548	2.578	.662

χ^2^ = 24.411; *df* = 5; p < .001; C.I = Confidence Interval

**Table 7:** Logistic regression analysis of demographic factors associated with environmental/occupational optometry.

Factors	Odds Ratio	95% C.I for Odds Ratio		p-value
		Lower Bound	Upper Bound	
Age	.942	.797	1.114	.487
Gender	.828	.406	1.689	.603
Institution	.864	.456	1.635	.652
Place of origin	1.136	.586	2.199	.706
Year of study	1.418	.691	2.912	.341
First choice programme	.538	.275	1.053	.070
Information on career opportunity	1.740	.597	5.074	.311
Source of career information	.793	.154	4.096	.782

χ^2^ = 3.442; *df* = 1; p = .064; C.I = Confidence Interval

**Table 8:** Logistic regression analysis of demographic factors associated with paediatric optometry.

Factors	Odds Ratio	95% C.I for Odds Ratio		p-value
		Lower Bound	Upper Bound	
Age	.991	.823	1.195	.928
Gender	2.234	1.191	4.191	.012
Institution	.698	.363	1.342	.281
Place of origin	1.157	.580	2.306	.680
Year of study	1.240	.617	2.493	.545
First choice programme	.845	.417	1.713	.641
Information on career opportunity	.578	.233	1.434	.237
Source of career information	.800	.185	3.456	.765

χ^2^ = 6.246; *df* = 1; p = .012; C.I = Confidence Interval

## Career-related information and sector of practice

Students graded their access to information about their career as very good, good, fair, poor, and very poor. Less than half (45.0%) of students assessed their level of access to information about career opportunities as good and 34.4%, 12.0%, 5.7%, 2.9% graded theirs' as fair, poor, very good and very poor respectively.

About a third of the students (33.5%) were of the view that their departments sometimes (once per two years) organize career seminars for them. Fifty-nine (28.2%), 19.6%, 14.4% and 4.3% were of the view that their departments organize career seminars rarely (once per three years), very often (once per year), never (none since year one) and always (once per semester) respectively. There was an association between frequency of career seminars and institution of study with UCC more likely to organize career seminars for optometry students (χ^2^ (4) = 21.658, p< .001).

Majority of the participants (53.6%) aspire to practice in the private sector (including Non-Governmental Organisation—NGO facilities) with the rest (46.4%) desiring to be in the public sector. Majority of the students (61.6%) from KNUST reported that they aspired to be in the private sector and 56.0% of the students from UCC preferred joining the public sector after completing their studies. There was an association between students’ institution of study and their aspired sector of practice (χ^2^ (1) = 15.382, p< .001).

## Discussion

To the authors best knowledge this is the first study in Ghana to evaluate career aspirations of optometry students and the second study to assess factors affecting career choices of optometry students. The majority (64.6%) of students aspire to be in clinical practice. About a third (28.9%) of students chose environmental/occupational optometry as the most desired optometric interest area. 26.8% of the students received career information from practicing optometrists. The most prevalent factors influencing students career choices were interest in career field (64.1%) and potential good income (38.3%).

This study confirms the findings from the previous study of low proportion of female students in optometry training institutions in Ghana [15]. This could be attributed to the general low proportion of female admissions into higher education in Ghana and the overall better performance of males than females in the West African Senior School Certificate Examination [20]. In contrast, a study from South Africa [21] reported a higher representation of females (69.5%) than males (30.5%) in the optometry programme in South Africa. This disparity may be from differences in national policy and cultural setting between the two countries. The majority (61.2%) of the students from urban areas reveal a worrying geographical disparity of students enrolled into the optometry programme in Ghana since approximately 49.1% of Ghanaians live in rural areas [22]. Considering the challenges with healthcare in rural Ghana and in light of the fact that students from rural origin are reported to have the greatest desire to practice in the rural setting [10], optometry admitting universities in Ghana may have to re-evaluate their policy. The two optometry schools may have to re-evaluate their admission policies towards recruiting more female students and those from rural areas into the optometry programme. In this regard, it is recommended that the optometry programme be specially included in award/scholarship and grant seeking projects targeted at enrolling female students and those from rural areas.

The study showed that two thirds (65.6%) of our participants had optometry as the initial programme of choice in their application to university. This result is slightly higher than as previously reported in Ghana (61.4%) [15] and South Africa (60.5%) [21], and could mean that the profession of optometry is becoming increasingly popular. However, human biology/medicine, being the highest on the list among those who did not choose optometry as their initial choice programme, is not surprising as similar findings have been reported among pharmacy students in Sierra Leone [23]. This, possibly, confirms the widespread admiration and prestige which medicine as a profession enjoys from the public. The relatively higher number of UCC students selecting optometry as their first programme of study could be due to the numerous undergraduate health science and engineering programmes available in KNUST compared to UCC, hence KNUST students have a large pool of programmes to choose from during their university applications.

The majority of the participants (64.6%) choosing clinical practice and academia/research (28.2%) as their aspired career field portend an apparent bright image for the prospects of optometry and eye services delivery in Ghana and Africa. This finding could be underpinned by another observation in the study that most of the participants had their primary source of information on career opportunities in optometry from optometrists, probably during their clinical attachment periods and consistent with literature [16,17]. It is refreshing to know that Ghanaian optometry students will like to be in clinical practice especially as students from rural communities may be willing to accept postings to rural areas to advance the call for Universal Eye Health in the Global Action Plan Initiative [10]. Academia/Research as the second highest career choice is encouraging for the advancement of optometry education in Africa because in recent years, Ghanaian trained optometrists have been engaged in the development of optometry programmes in universities in African countries such as Malawi and Kenya. Binary logistic regression showed that students who received good/very good information on career opportunities in academia/research were five times more likely to have interest in that field, suggesting the need to provide adequate information to students on various career opportunities. Females were twice more likely to practice optometry and pursue an interest in paediatric optometry than males in our study, this finding further emphasizes the need to admit more females into the optometry programme. Interestingly, Business Administration, Law and Fashion designing were among the numerous career fields mentioned by participants in this study, suggesting that the optometry programme and its training in Ghana is versatile and holistic; hence optometry students in Ghana may be more open to pursue their talents and interests. The varied responses on the optometric interest areas among participants could be explained by the impressive level of knowledge and appreciation of the existing opportunities available in optometry among the students as observed in this study. This could translate into having many optometrists with varied interest areas in Ghana and Africa in the future. Binary logistic regression analysis revealed that none of the students’ demographic characteristics used in the analysis were significantly associated with environmental/occupational optometry as an optometric interest area.

Contrary to reports that ‘job availability after graduation’ and ‘desire to help others’ were the main factors which influenced career choices of optometry students in Ghana and South Africa, this study found interest in optometric practice as the main driving force for career choices among optometry students in Ghana [15,21]. This suggests that the optometry profession is becoming increasingly popular among students in Ghana. Nonetheless, potential good income, flexible working hours and job availability were other factors mentioned by students as influencing their career choices and are consistent with these previous studies. It is worth noting that contrary to a finding of high parental influence on career choices in the USA [19], this study found very little or no parental influence on career choices, possibly suggesting the independent mindedness of the participants.

There were no significant associations between career aspirations and participants’ institution of study as well as their choice of optometry or otherwise as an initial programme of study during application to the university. This could mean that no significant differences in training exist for optometry students in Ghana regardless of their institution of training. It could also be explained by the fact that most of these students receive information from the same optometrists during their clinical attachment and externship in the various eye clinics in Ghana.

The majority of participants (53.6%) aspiring to practise in the private sector/NGOs in light of reports that many optometrists in Ghana were found to be in the public sector [11], seemingly presents a new direction for the future of optometry in Ghana; the impact of this on the profession of optometry may have to be explored in a future study.

The high response rate (97%) and the prospective nature of the study means that the findings of this study reflects Ghanaian optometry students career aspirations. However, a major limitation was our inability to determine if their career choices changed over the years and also which career options they considered a priority.

## Conclusions

Optometry students in Ghana largely aspire to be in clinical practice and that the greatest influence on their career choices was a psycho-social motivation or personal factor (interest in career field). Also, practical motivations such as potential good income, flexible working hours and job availability were major influences on career choice. Factors such as student’s choice of optometry as an initial programme of study during university application have no influence on optometry students career aspirations in Ghana. It is recommended that the optometry programme be specially included in award/scholarship and grant seeking projects targeted at enrolling female students and those from rural areas.

## Supporting information

S1 Table Data collection form. (DOCX)

S1 Fig Ethical approval. (DOCX)

S1 File Dataset. (SAV)
