# Supplementary material for: Career aspirations and factors influencing career choices of optometry students in Ghana
Source: PLoS One. 2020 May 29;15(5):e0233862. doi: 10.1371/journal.pone.0233862 (PMC7259716; doi:10.1371/journal.pone.0233862)
Supplement: S1 Table — (DOCX) [file pone.0233862.s001.docx]

**S1 Table: Data Collection Form**

**CAREER ASPIRATIONS OF OPTOMETRY STUDENTS IN GHANA**

**QUESTIONNAIRE FOR PARTICIPANTS**

**Kindly indicate the number attached to the answer option as your answer and tick answer boxes where necessary**

| **S/No:** | **DEMOGRAPHIC DATA DATE:** | **ANSWER OPTIONS** | **ANSWER** |
| --- | --- | --- | --- |
| 1. | Study Number |  | |
| 2. | Age | years | |
| 3. | Gender | 0= Male 1= Female |  |
| 4. | Nationality | 0= Ghanaian 1= Non-Ghanaian |  |
| 5. | Place of origin  **[Ghanaian students only]** | 0= Rural 1= Urban |  |
| 6. | Region of origin  **[Ghanaian students only]** | 0= Ashanti 1= Northern  2= BA 3= U/E  4= Eastern 5= U/W  6= GAR 7= Western  8= Volta 9= Central |  |
| 7. | School | 0= KNUST  1= UCC |  |
| 8. | Year of study | 0= Four  1= Five  2= Six |  |

| **S/No:** | **CHOICE OF OPTOMETRY PROGRAMME** | **ANSWER OPTIONS** | **ANSWER** |
| --- | --- | --- | --- |
| 9. | What was your first programme of choice when seeking admission into the university? | 0= Optometry 1= Pharmacy  2= Medicine 3= Engineering  4= Dentistry 5= Med Lab  6= Other; please specify |  |

| **S/No:** | **CAREER ASPIRATIONS** | **ANSWER OPTIONS** | **ANSWER** |
| --- | --- | --- | --- |
| 10. | How well are you informed on the career opportunities in optometry available after graduation? | 0= Very poor  1= Poor  2= Fair  3= Good  4= Very good |  |
| 11. | Please what was your **primary source** of information in **(10)** above? | 0= Lecturers  1= Senior course mates  2= Internet/Online  3= Optometrists  4= Seminars  5= Family/Friends  6= other; please specify |  |
| 12. | How often does your department organise career seminars for you? | 0= Never [None since year one]  1= Rarely [once per 3 years]  2= Sometimes [once per 2 years]  3= Very often [once per year]  4= Always [once per semester] |  |
| 13. | Which career field(s) do you aspire to pursue? | - Clinical practice - Academia/Research - Industrial optometry [eg. Ophthalmic and optical device companies] - Public Health Optometry - Non-Optometric [eg. Law, Business administration, Military, etc] ; please specify - Uncertain   **[Tick all boxes that apply]** |  |
| 14. | What factor(s) influenced your choice(s) in (**13**) above? | - Potential good income - Job availability - This is what my parents want me to do - This is where my interest lies - Flexible working hours - This is the only thing I know of - Other; please specify   **[Tick all boxes that apply]** |  |
| 15. | Where do you aspire to practice? | 0= Public  1= Private (including NGOs) |  |
| 16. | Which optometric speciality area(s) do you aspire to pursue? | - Contact lens - Low vision - Paediatric optometry - Geriatric optometry - Environmental/Occupational optometry - Binocular vision - Uncertain - Other; please specify   **[Tick all boxes that apply]** |  |
