# Supplementary figures and images for: Career aspirations and factors influencing career choices of optometry students in Ghana
Source: PLoS One. 2020 May 29;15(5):e0233862. doi: 10.1371/journal.pone.0233862 (PMC7259716; doi:10.1371/journal.pone.0233862)

**S1 Fig: Ethical Approval**

**
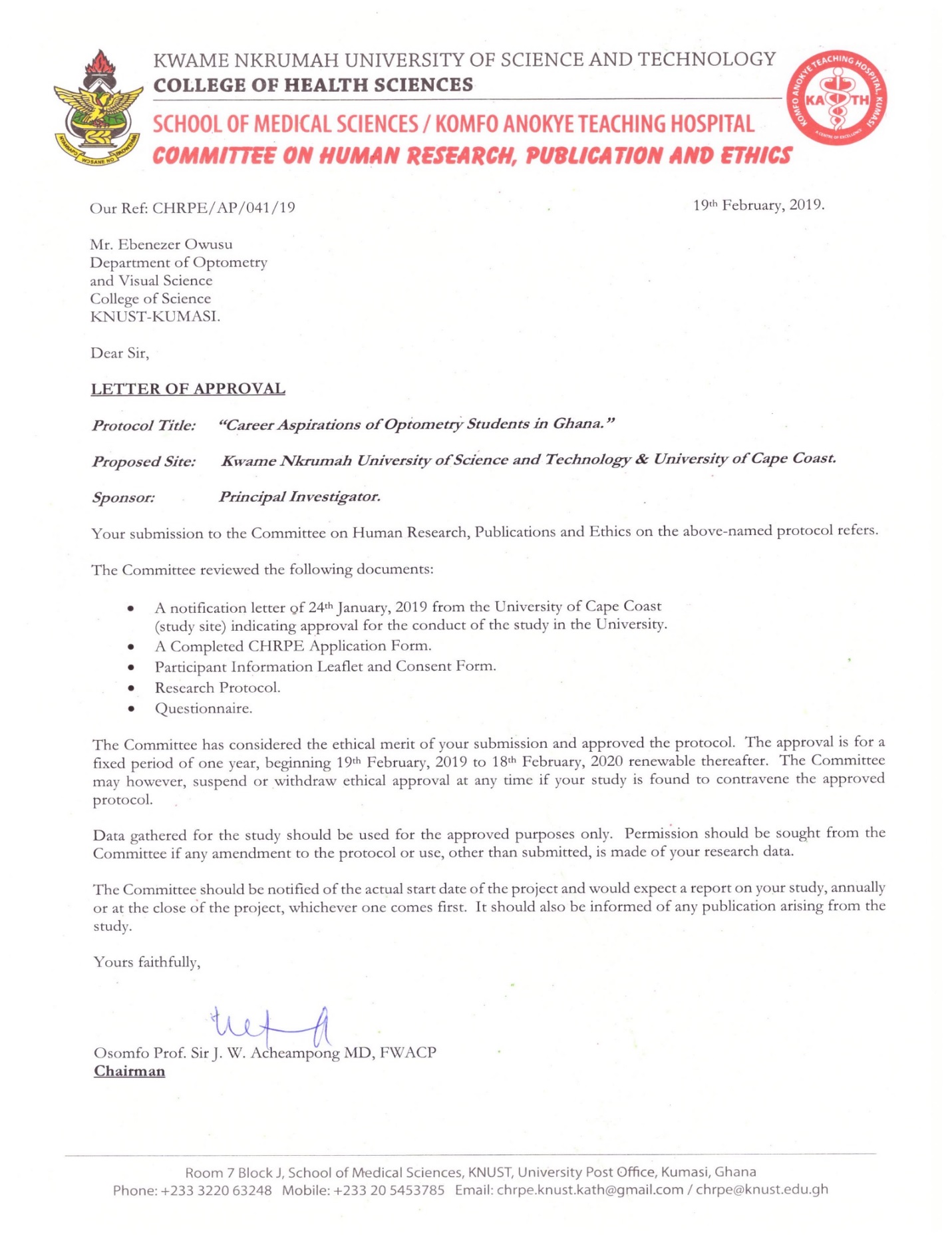
**

Supplement: S1 Fig — (DOCX) [file pone.0233862.s002.docx]
